# Jumping in aquatic environment after sciatic nerve compression: nociceptive evaluation and morphological characteristics of the soleus muscle of Wistar rats

**DOI:** 10.1590/S1679-45082017AO3613

**Published:** 2017

**Authors:** Jéssica Aline Malanotte, Camila Mayumi Martin Kakihata, Jhenifer Karvat, Rose Meire Costa Brancalhão, Lucinéia de Fátima Chasko Ribeiro, Gladson Ricardo Flor Bertolini

**Affiliations:** 1 Universidade Estadual do Oeste do Paraná, Cascavel, PR, Brazil.

**Keywords:** Sciatic nerve/pathology, Muscle, skeletal, Physical therapy modalities, Pain, Rats, Wistar

## Abstract

**Objective:**

To evaluate the effect of jumping in aquatic environment on nociception and in the soleus muscle of trained and not trained Wistar rats, in the treatment of compressive neuropathy of the sciatic nerve.

**Methods:**

Twenty-five Wistar rats were distributed into five groups: Control, Lesion, Trained + Lesion, Lesion + Exercise, and Trained + Lesion + Exercise. The training was jumping exercise in water environment for 20 days prior to injury, and treatment after the injury. Nociception was evaluated in two occasions, before injury and seven after injury. On the last day of the experiment, the right soleus muscles were collected, processed and analyzed as to morphology and morphometry.

**Results:**

In the assessment of nociception in the injury site, the Control Group had higher average than the rest, and the Lesion Group was larger than the Trained + Lesion and Lesion + Exercise Groups. The Control Group showed higher nociceptive threshold in paw, compared to the others. In the morphometric analysis, in relation to Control Group, all the injured groups showed decreased muscle fiber area, and in the Lesion Group was lower than in the Lesion + Exercise Group and Trained + Lesion Group. Considering the diameter of the muscle fiber, the Control Group had a higher average than the Trained + Lesion Group and the Trained + Lesion + Exercise Group; and the Lesion Group showed an average lower than the Trained + Lesion and Lesion + Exercise Groups.

**Conclusion:**

Resistance exercise produced increased nociception. When performed prior or after nerve damage, it proved effective in avoiding hypotrophy. The combination of the two protocols led to decrease in diameter and area of the muscle fiber.

## RESUMO

**Objetivo:**

Avaliar os efeitos do salto em meio aquático, na nocicepção e no músculo sóleo, em ratos Wistar treinados e não treinados, no tratamento de neuropatia compressiva do nervo isquiático.

**Métodos:**

Foram distribuídos em cinco grupos 25 ratos Wistar: Controle, Lesão, Treinado + Lesão, Lesão + Exercício e Treinado + Lesão + Exercício. O treino foi com exercício de salto em meio aquático durante 20 dias, prévio à lesão, e o tratamento ocorreu após a lesão. Foram realizadas avaliações da nocicepção, sendo uma pré-lesão e sete pós-lesão. No último dia de experimento, os músculos sóleos direitos foram coletados, processados e analisados por meio de morfologia e morfometria.

**Resultados:**

Na avaliação da nocicepção no local da lesão, o Grupo Controle apresentou média maior que os demais, e o Grupo Lesão foi maior que os Grupos Treinado + Lesão e Lesão + Exercício. O Grupo Controle apresentou limiar nociceptivo na pata maior com relação aos demais. Nas análises morfométricas, em relação ao Grupo Controle, todos os grupos lesionados apresentaram diminuição da área da fibra muscular; o Grupo Lesão apresentou-se menor que os Grupos Treinado + Lesão e Lesão + Exercício. No diâmetro da fibra muscular, o Grupo Controle apresentou média maior que os Grupos Treinado + Lesão e Treinado + Lesão + Exercício, e o Grupo Lesão apresentou média menor que os Grupos Treinado + Lesão e Lesão + Exercício.

**Conclusão:**

O exercício físico resistido produziu aumento da nocicepção. Quando realizado previamente ou após a lesão nervosa, mostrou-se eficaz em evitar a hipotrofia. A associação dos dois protocolos levou à diminuição do diâmetro e da área da fibra muscular.

## INTRODUCTION

Peripheral nerve disorders are common conditions in clinical practice.^1^ They directly affect muscle functions, because they may interrupt neuromuscular communication,^2^ trigger several phenomena that lead to degeneration, and negatively affect its functionality and structure. Among the alterations in morphology are an increase in intramuscular connective tissue and a disarrangement and atrophy of the muscle, evidenced by a decrease in the cross-sectional area of the muscle and the muscle fibers.^3^ In addition, peripheral nerve lesions (PNL) result in pain along the nerve pathway and reduced or lost sensitivity and motricity at the innervated site, which, in turn, result in functional limitations.^4,5^

There are several forms of treatment, including surgical interventions, which have relatively poor functional outcomes, and non-surgical therapeutic approaches.^2^ Among these, physical exercise after injury stands out, to avoid functional losses during nerve regeneration.^6^ Muscle fibers are highly adaptable, depending on the stimuli used, as these can alter their metabolism and size; prevent the effects of muscle denervation; improve neuromuscular transmission; and potentiate neuroplasticity.^7^

The morphological adaptations caused by movement occur in response to physical stress stimulus. Muscle tissue can increase the contraction strength and oxygenation of cellular sarcoplasm, thus creating calcium ion (Ca^++^) and adenosine triphosphate (ATP) storage mechanisms.^8^

To minimize PNL-induced muscular atrophy, the use of resisted physical exercise, such as aquatic jump, prior to nerve injury is a suggested therapy.^9^ This exercise, although indicated, still has some unanswered gaps, such as a scientific evidence of its efficacy in peripheral neuropathies.^10^

Therefore, physical exercise after nerve injury can have no effect at all, or may even present poor muscle and functional outcomes. However, there are very few studies in the literature on muscle treatment models in animals with pre-injury physical training and the association between the two protocols.^7,8,11^ It is interesting to evaluate exercises for the soleus muscle, which is predominantly composed of oxidative fibers,^12^ and is innervated by the sciatic nerve, which has a validated compression model.^13^

## OBJECTIVE

To evaluate the effects of aquatic jump on nociception and the soleus muscle, in trained and untrained Wistar rats, in the treatment of sciatic nerve compressive neuropathy.

## METHODS

This study was conducted according to the International Norms of Ethics in Animal Experimentation, approved by the Committee of Ethics in Animal Use (CEUA) of the *Universidade Estadual do Oeste do Paraná* (*Unioeste*), and carried out from February to June 2015 in this institution.

A total of 25 male Wistar rats, 8 weeks old, mean weight of 314±23g, exposed to a 12-hour/12-hour light-dark photoperiod, at a temperature of 23°C, with free access to water and food, were used in this study.

### Sample groups

The animals were even and randomly distributed in five groups:

- Control Group (C): no intervention, the animals were free to move in the cage during the whole experiment, and were euthanized on the 42^nd^ day of the experiment.

- Lesion Group (L): the animals were subjected to nerve compression on the 21^st^day of the experiment. They were free to move in the cage until euthanasia on the 42^nd^ day of the experiment, or 21 days after surgery.

- Trained + Lesion Group (TL): the animals exercised three times a week for 20 days. Later, they were subjected to nerve compression on the 21^st^ day of the experiment, and euthanized 21 days after surgery.

- Lesion + Exercise Group (LE): the animals were subjected to nerve compression on the 21^st^ day of the experiment, and then performed resisted exercise three times a week for 20 days. They were euthanized 21 days after surgery.

- Trained + Lesion + Exercise Group (TLE): the animals performed resisted exercise three times a week for 20 days. They were subjected to nerve compression on the 21^st^day of the experiment, then they performed resisted exercise three times a week for 20 days, and finally, they were euthanized 21 days after surgery.

### Exercise protocol

**Figure 1:**
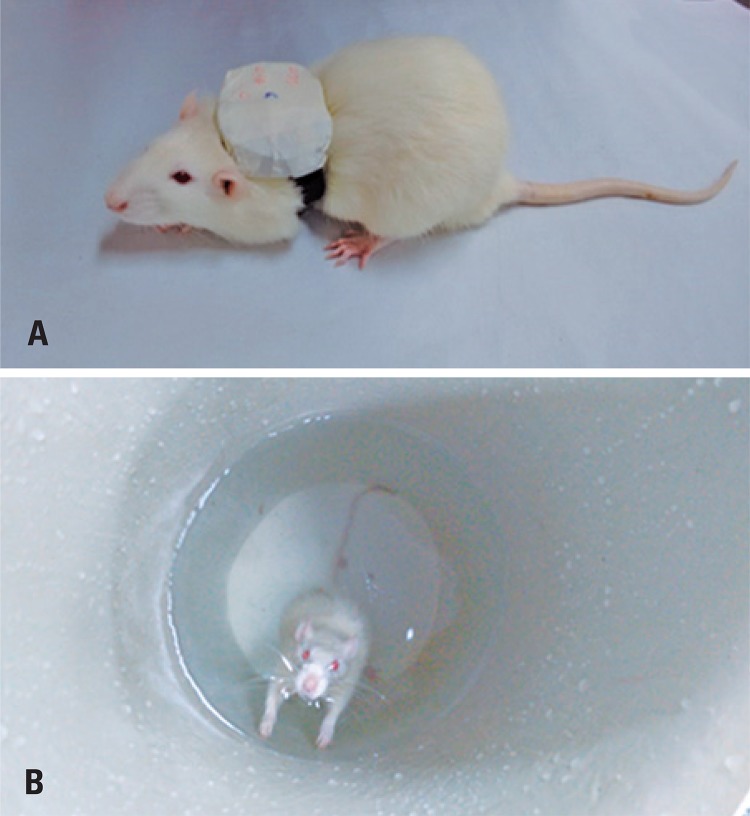
(A) Animal with overload weight attached with velcro strap. (B) Performing the jumping exercise in aquatic environment

The protocol consisted of jumping exercises in aquatic environment, with the animal placed in a 30cm diameter and 55cm high cylindrical tube, inside a 200L water tank, at a temperature of 33°C±1. A 50% body weight overload was attached with a Velcro^®^ strap to the back of the animal (Figure 1A), which caused the animal to submerge and, upon reaching the bottom of the tank, to leap upward to reach the surface (Figure 1B). Each impulse was counted as a jump.^9^

The jump exercise protocol was performed for 20 days, with 3 days of exercise followed by a one-day interval each week, and a two-day interval between weeks. In the first week, two sets of ten jumps were performed; in the second week, three sets of ten jumps; and in the third week, four sets of ten jumps, with 30 second intervals between sets. The pre-lesion training protocol and the post-lesion exercise protocol were similar. The difference was that after the lesion, the exercise was started on the third postoperative day.

### Experimental model of sciatic nerve compression

Before surgical sciatic nerve compression, the animals were weighed and intraperitoneally anesthetized with ketamine hydrochloride (80mg/kg) and xylazine hydrochloride (40mg/kg). Afterwards, an incision was made to expose the right sciatic nerve and, then, the nerve was crushed with a hemostatic forceps, for 30 seconds. The clamping pressure was standardized for all animals, using the second tooth of the rack as a reference, and all clamping procedures were done by the same individual.^13^ The animal was then sutured with 4.0 Catgut sutures.

### Evaluation of nociception

To evaluate nociception, an Insight^®^ (Ribeirão Preto, São Paulo, Brazil) von Frey Hair digital aesthesiometer was used. The equipment consists of a handle with a disposable polypropylene probe, capable of evaluating a force range of 0.1-1000g, connected to an amplifier speaker.^14^ The filament was applied to the site of the sciatic nerve lesion and the plantar region of the animal’s right pelvic limb. To exert pressure on the lesion site, the animal was manually restrained, and the filament tip contacted the lesion site until the animal removed its limb. To exert pressure on the right plantar region, the animal was kept in a raised box with a floor grid. The filament was then positioned, and pressure was exerted until the animal removed its right limb. The evaluation periods are described in chart 1.

**Chart 1:** Evaluation periods of the digital aesthesiometer

Evaluation	Period
1	Pre-lesion
2	3^rd^ postoperative day before exercise in LE and TLE Groups
3	3^rd^ postoperative day after exercise in LE and TLE Groups
4	7^th^ postoperative day after exercise in LE and TLE Groups
5	10^th^ postoperative day before exercise in LE and TLE Groups
6	14^th^ postoperative day after exercise in LE and TLE Groups
7	17^th^ postoperative day before exercise in LE and TLE Groups
8	21^st^ postoperative day after exercise in LE and TLE Groups

LE: Lesion + Exercise; TLE: Trained + Lesion + Exercise.

### Histomorphometric analysis

On the last day of the experiment, the animals were anesthetized and euthanized by guillotine decapitation. Then, the right soleus muscle was dissected, sectioned along a transversal plane, fixed in Metacarn for 2 hours, and stored in 70% alcohol until the histological procedure. The muscles were dehydrated in a series of increasing alcohol concentrations, diaphanized, infiltrated, and embedded in histological paraffin, to obtain 7μm transverse cut slides.

The slides were stained with hematoxylin and eosin (HE), photomicrographed in ten fields per muscle, and analyzed for the smallest diameter and area in one hundred fibers per muscle, using Image-Pro Plus 6.0 software. The muscles were also stained in Masson trichrome, to quantify connective tissue, using a pixel counting system (photomicrographs taken with a 40x objective), and they were also analyzed using Image-Pro Plus 6.0 software. The relative area of the connective tissue was calculated by rule of three.

### Data analysis

The results were expressed and analyzed using descriptive and inferential statistics. Data were first evaluated for their normality by the Shapiro-Wilk test and, since they presented a normal distribution, a univariate analysis of variance (ANOVA) was used for the histomorphometric analyzes, with a *t* post-test, when there was a significant difference. For the functional analyzes, a mixed ANOVA model was used. It was considered significant when p<0.05. The results were expressed by the F statistic and mean.

## RESULTS

### Nociception at the lesion site

**Figure 2:**
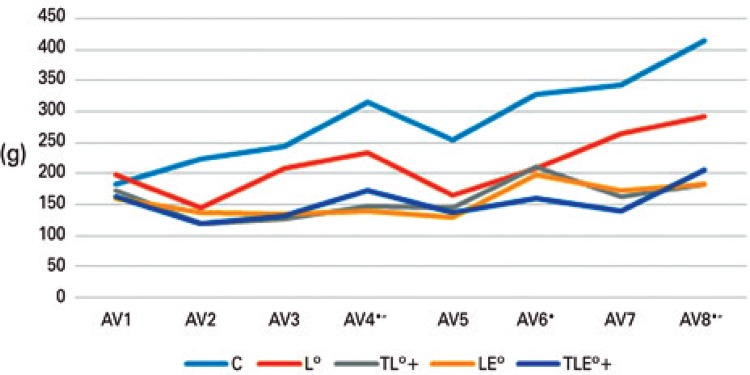
Evaluation of nociception at the animal’s lesion site

There were significant differences among the evaluations (F[95.539;3.981]=52.352; p<0.001). The evaluations 1 to 3 were lower than the evaluation 4 and the evaluations 6 to 8 (p<0.001); and the evaluation 5 was lower than the evaluations 4 to 8 (p<0.001), evidencing a threshold increase effect by the sum of evaluations, visible mainly for the Control Group. There were also differences among the groups. The Control Group was higher than the others, and the Lesion Group was higher than the Trained + Lesion and the Lesion + Exercise Groups (p<0.001), showing an increased nociception when exercise was performed before or after the lesion (Figure 2).

* Difference from AV1; “ Difference from AV3; ° Difference from Controle Group (symbols presented next to the abbreviations).

C: Control Group; L: Lesion Group; TL: Trained + Lesion Group; LE: Lesion + Exercise Group; TLE: Trained + Lesion + Exercise Group.

### Nociception in the paw

**Figure 3:**
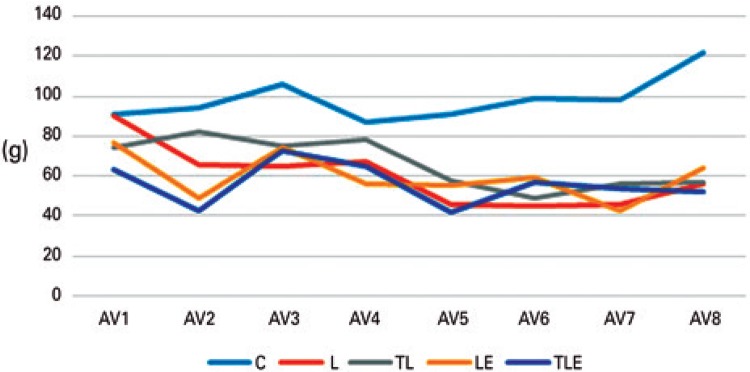
Evaluation of nociception in the animal’s paw

In the evaluation of nociception in the paw, there were significant differences among the evaluations [F(7;168)=7.256; p<0.001). The evaluation 1 was higher than the evaluations 5 to 7 (p<0.001), and the evaluation 3 was higher than the evaluation 5 (p=0.001) and 7 (p=0.024). For this form that aimed to evaluate the allodynia, there was progressive worsening of the variable. There were also differences among the groups [F(168;28)=2.557; p=0.001]. The Control Group had a higher mean than the others (p<0.001), and the lesion groups had a similar behavior (Figure 3).

° Difference from Control Group.

C: Control Group; L: Lesion Group; TL: Trained + Lesion Group; LE: Lesion + Exercise Group; TLE: Trained + Lesion + Exercise Group.

### Muscle fiber area

**Figure 4:**
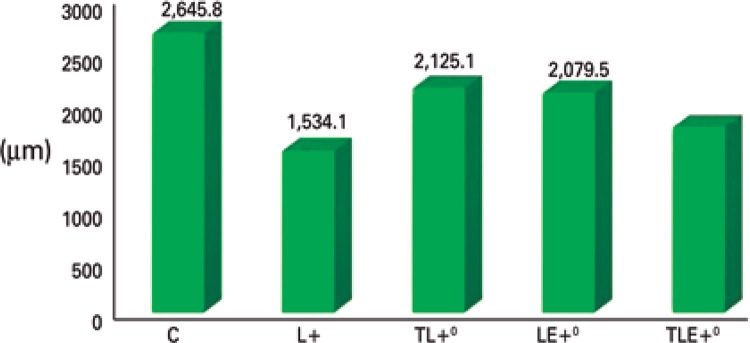
Muscle fiber area

There were significant differences among the groups [F(4;20)=7.4105; p=0.001]: among the Control Group and Lesion Group (p<0.001), Trained + Lesion Group (p=0.0275), Lesion + Exercise Group (p=0.0176), Trained + Lesion + Exercise Group (p<0.001). The Control Group was higher than the others. There were also differences among the Lesion Group and the Trained + Lesion (p=0.0138) and the Lesion + Exercise (p=0.0216) Groups. The Lesion Group was lower then the Trained + Lesion Group and the Lesion + Exercise Group, indicating greater atrophy in the group with only the lesion (Figure 4).

+ Difference among C group and L and TLE groups; ° Difference among L group and TL and LE groups.

C: Control Group; L: Lesion Group; TL: Trained + Lesion Group; LE: Lesion + Exercise Group; TLE: Trained + Lesion + Exercise Group.

### Muscle fiber diameter

**Figure 5:**
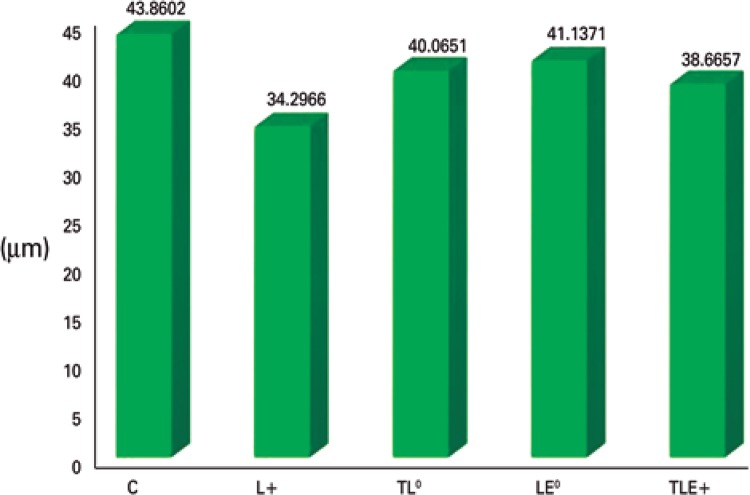
Muscle fiber diameter

In the fiber diameter, there were statistically significant differences (F[4;20]=4.2165; p=0.0123) among the Control Group and the Lesion (p<0.001) and the Trained + Lesion + Exercise (p=0.0449) groups. The Control Group had a higher mean than the others. There were also differences among the Lesion Group and the Trained + Lesion (p=0.0276) and the Lesion + Exercise (p=0.0106) groups, and the Lesion Group was lower than the others, confirming the finding that for this group the atrophy was more intense (Figure 5).

+ Difference among the Control Group and the Lesion and the Trained + Lesion + Exercise Groups; ° Difference among the Lesion Group and the Trained + Lesion and the Lesion + Exercise Groups.

C: Control Group; L: Lesion Group; TL: Trained + Lesion Group; LE: Lesion + Exercise Group; TLE: Trained + Lesion + Exercise Group.

### Connective tissue

**Figure 6:**
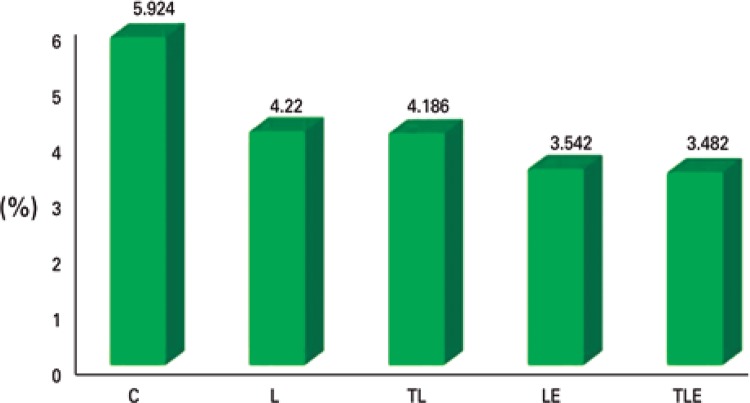
Evaluation of connective tissue

In the evaluation of the connective tissue, there were no significant differences (F[4;20]=1.2113; p=0.0337] (Figure 6).

C: Control Group; L: Lesion Group; TL: Trained + Lesion Group; LE: Lesion + Exercise Group; TLE: Trained + Lesion + Exercise Group.

### Morphological analysis of muscle tissue

**Figure 7:**
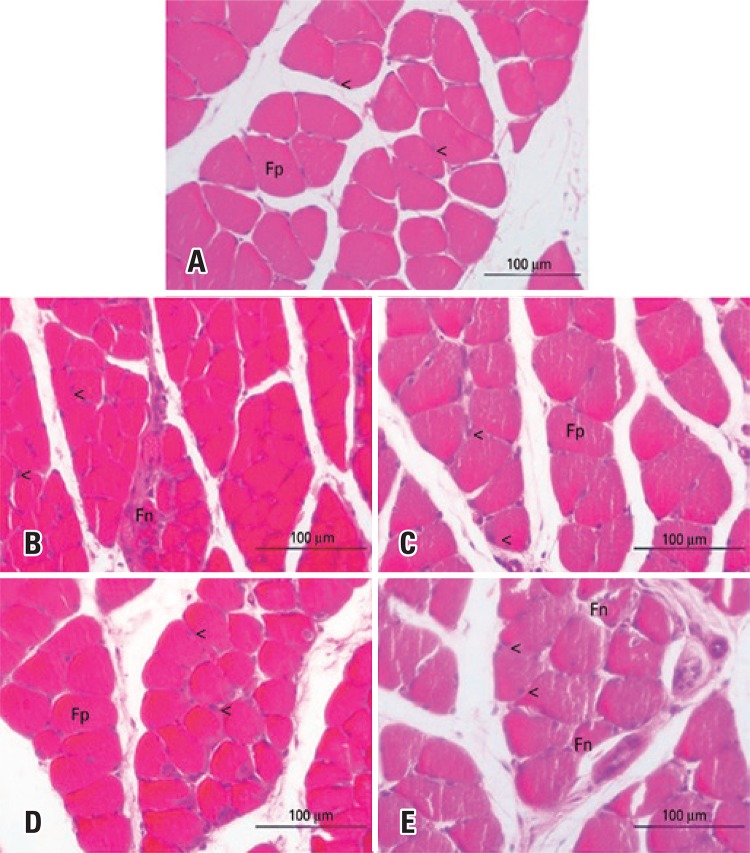
Photomicrographs of the right soleus muscle, transverse section, stained with hematoxylin and eosin. (A) Control Group; (B) Lesion Group; (C) Trained + Lesion Group; (D) Lesion + Exercise Group; (E) Trained + Lesion + Exercise Group

In the Control Group, the morphological analysis of the right soleus muscle showed polygonal muscle fibers, nuclei in the peripheral position, and normal fascicular pattern (Figure 7A). The Lesion Group (Figure 7B) showed a large amount of polymorphic fibers, with preservation of nuclear position and presence of fibers with morphological alteration. In the Trained + Lesion and the Lesion + Exercise Groups, most muscular fibers returned to their characteristic polygonal format, the nuclei were in peripheral position (Figures 7C and 7D), and no fibers with morphological alteration were found. In the Trained + Lesion + Exercise Group, there was a large number of polymorphic fibers (Figure 7E), the nuclei maintained their peripheral positioning, and it was possible to observe the presence of fibers with morphological alterations.

## DISCUSSION

The practice of physical exercises has been gaining more prominence because it is effective both in prevention and treatment of injuries.^15^ One of the modalities is resisted physical exercise, considered one of the most efficient methods to increase strength and muscle mass,^16^ in addition to promoting analgesia.^17^ Based on several protocols, therapeutic exercises have been used to alleviate PNL symptoms.

In nerve injury, neutrophil invasion and concentration occur at the lesion site in 24 to 72 hours, besides the recruitment of histamine releasing inflammatory cells.^18^ Pro-inflammatory cytokines, such as interleukin (IL) 1beta, IL-6, and tumor necrosis factor alpha (TNF-α), stimulate a cascade of events that sensitizes nociceptors,^19^ resulting in prolonged hyperalgesia, due to an increase in the excitability of neurons and the induction of adrenergic sprouting.^20^

Performing resisted exercises results in greater neuromuscular activation and dispersion of acetylcholine receptors in the end plate, by stimulating the increase of the neuromuscular junction diameter.^21^ These alterations improve muscle strength and help maintain the effects of muscle contraction, even after cessation of training. In addition, the practice of regular physical exercises, as in the case of athletes, leads to an improvement in hormonal balance and a decrease in central sensitization, with a consequent reduction of pain.^22^ It is important to perform resisted exercises after the lesion, because this induces analgesia, possibly by increasing the pain threshold and the level of endogenous opioids. The duration of this analgesia may vary according to the intensity, duration, and type of exercise.^15^

In the present study, no analgesic effect of exercise was observed. The trained groups showed a decrease in pain threshold in the animals that performed exercises before the lesion. The duration of training may not have been enough to bring about these adaptations. Additionally, these effects were also not observed in the groups that performed the exercise after the lesion. This may be due to the initiation of exercise on the third postoperative day, when the concentration of neutrophils and inflammatory cells is increased at the lesion site. The exercise may also have caused hypernociception due to skin friction at the lesion site,^22^ since, in neuropathic pain, skin sensitivity is increased. Furthermore, the gluteal and femoral biceps muscles were sensitive because of reflection in surgery, impairing tissue healing. Also, the type of exercise chosen (resisted) can produce late-onset muscle soreness.

These results corroborate those of Gaffuri et al.,^9^ who evaluated the effectiveness of physical exercise, using swimming and jumping, in the pain pattern of rats subjected to sciatica. Treatment with physical exercise, with or without overload, was not effective in reducing pain. An intense physical training in rats with sciatic lesions induced an increase in the tolerance of opioid receptors, generating pain exacerbation.

Exercise in aquatic environment rely on some physical properties of water, such as heat and buoyancy, which can block nociception due to the effect of thermal conditions, since the heat of the water increases blood flow, aiding in the dissipation of pain-inducing catabolites.^9^However, it can be assumed that the temperature also influenced the absence of analgesic results, because in the initial phase of inflammation, an increase in local temperature can enhance the inflammatory process, leading to increased nociception.^23^

There was a decrease in muscle fiber area in all the lesion groups, but performing exercises before or after the lesion had positive results in trophism and muscle fiber area, which were greater than in the Lesion, the Trained + Lesion, and the Lesion + Exercise Groups, demonstrating that exercise can respectively prevent and reverse this nerve damage effect. There was a decrease in diameter in the lesion group and in the group that associated the two protocols (Lesion and Trained + Lesion + Exercise). In the Trained + Lesion and the Lesion + Exercise Groups, which performed exercises before or after the lesion, there was a restoration of the muscle fiber diameter and area.

This result may have been due to the fact that regular exercise promotes an antioxidant protection of muscle cells, or else, intense exercise increases some proteins, which contribute to the restoration of the protein homeostasis of these muscle fibers.^7^ Therefore, the expression of these proteins may have therapeutic effects, contributing to the protection against muscular atrophy and degeneration in periods of disuse. In this study, resisted exercises with weight load generated an increase in muscle mass to the point of minimizing atrophy due to PNL in the Trained + Lesion and the Lesion + Exercise Groups.

Bonetti et al.,^24^ while testing balance and coordination and endurance training, in order to accelerate regeneration after sciatic nerve crushing in rats, analyzed nerve and muscle morphology, and obtained better performance results in the tests with trained animals, and significantly larger muscle area than in untrained animals. Their results indicate that training in the initial phase after PNL improves the morphological properties of the soleus muscle and nerve, similar to what was observed in the present study: improvement in muscle fiber area and diameter in the Lesion + Exercise Group, who performed the training right after the nerve lesion.

However, the association of these protocols in the Trained + Lesion + Exercise Group led to worse results, such as decreased muscle fiber diameter and area, which may have occurred due to overtraining*.* An imbalance between stress and recovery can generate a process of excessive stress loads in training, combined with insufficient recovery time, with a decline in performance.^25^ The intensity and volume of training may have exceeded the body’s recovery and adaptation capacity, leading to muscle lesion, generating muscular atrophy in the Trained + Lesion + Exercise Group.

Muscle fibers have an intimate relation with the connective tissue, which is an extracellular matrix that surrounds muscle fibers and is important for the maintenance of the integrity and the properties of the muscle in the production of movement and force. The impairment of muscular innervation affects the connective tissue, evidenced by its intramuscular increase. If denervation remains for a long period, fibrous connective tissue replaces the contractile elements of the muscle, inhibiting muscle regeneration.^26^ However, these alterations were not found in this study. This may be justified by the duration of the study, which was not sufficient to induce these potential intramuscular connective tissue changes.

As limitations of the study, we highlight the absence of an evaluation of inflammatory markers, and performing resisted exercises for a longer period before and after the lesion, to observe the effects on nociception and on the connective tissue, respectively. We suggest these matters may be subjected to further investigation in the future. Furthermore, the small size of the sample can be considered another limitation. However, in animal experiments, due to the principle of reduction for ethical reasons and the design of the study, even when analyzing lesion models, the use of small samples^27^ may be acceptable in studies regarding the skeletal muscle tissue.^28,29^

## CONCLUSION

Resisted physical exercise was effective in the experimental model of nerve compression, leading to increased nociception. When performed before or after peripheral nerve lesion, resisted physical exercise was effective in avoiding atrophy of the soleus muscle, and induced an improvement in the general morphology of the muscle tissue and its morphometric parameters. Therefore, the study results highlight the importance of physical exercise as a preventive and rehabilitative factor in peripheral nerve lesions.

## References

[1] Bobinski F, Martins DF, Bratti T, Mazzardo-Martins L, Winkelmann-Duarte EC, Guglielmo LG (2011). Neuroprotective and neuroregenerative effects of low-intensity aerobic exercise on sciatic nerve crush injury in mice. Neuroscience.

[2] Possamai F, Siepko CM, André ES (2010). Investigation of therapeutic exercise effects on peripheral nerve regeneration. Acta Fisiatr.

[3] Caierão QM, Betini J, Teodori RM, Minamoto VB (2008). The effect of time interval between electrical stimulation on the denervated rat muscle. Rev Bras Fisioter.

[4] Mallmann JS, Moesch J, Tomé F, Vituri RF, Carvalho AR, Bertolini GR (2012). Muscular hypotrophy prevention by using neural mobilization in experimental sciatica model. Braz J Exerc Physiol.

[5] Pinto AC, Macea JR, Pecoraro MT (2012). Femoral nerve neuropathy after the psoas hitch procedure. einstein (São Paulo).

[6] Cunha NB, Ilha J, Centenaro LA, Lovatel GA, Balbinot LF, Achaval M (2011). The effects of treadmill training on young and mature rats after traumatic peripheral nerve lesion. Neurosci Lett.

[7] Artifon EL, Silva LI, Ribeiro LF, Brancalhão RM, Bertolini GR (2013). Aerobic training previous to nerve compression: morphometry analysis of muscle in rats. Rev Bras Med Esporte.

[8] Moret DG, Castoldi RC, Araújo RG de, Spagnol AR, Papoti M, Camargo JC (2013). Morphological analysis of muscle of rats submitted to a protocol of concurrent training. Rev Bras Ciênc Esporte.

[9] Gaffuri J, Meireles A, Rocha BP, Rosa CT, Artifon EL, Silva LI (2011). Physical exercise assessment as an analgesia factor in a sciatica experimental model. Rev Bras Med Esporte.

[10] Martínez de Albornoz P, Delgado PJ, Forriol F, Maffulli N (2011). Non-surgical therapies for peripheral nerve injury. Br Med Bull.

[11] Spagnol AR, Malheiro OC, Castoldi RC, Moret DG, Araújo RG, Papoti M (2012). Plasticity analysis muscle in rats submitted to one concurrent physical training protocol. Rev Bras Ciênc Mov.

[12] Piovesan RF, Martins MD, Fernandes KP, Bussadori SK, Selistre-de-Araújo HS, Mesquista-Ferrari RA (2009). Review on the plasticity of skeletal muscle: expression of myosin heavy chain isoforms and functional correlation. Fisioter Mov.

[13] Bridge PM, Ball DJ, Mackinnon SE, Nakao Y, Brandt K, Hunter DA (1994). Nerve crush injuries--a model for axonotmesis. Exp Neurol.

[14] Beyreuther B, Callizot N, Stöhr T (2007). Antinociceptive efficacy of lacosamide in the monosodium iodoacetate rat model for osteoarthritis pain. Arthritis Res Ther.

[15] Antunes JS, Karvat J, Meireles A, Rocha BP, Thieimi C, Silva LI (2012). Resistance exercise in water for Wistar rats submitted to tendinous trauma: nociception and edema assessment. Rev Dor.

[16] Cardoso EA, Bottaro M, Rodrigues P, Rezende CB, Fischer T, Mota J (2014). Chronic effects of resistance exercise using reciprocal muscle actions on functional and proprioceptive performance of young individuals: randomized controlled trial. Rev Bras Cineantropometria Desempenho Hum.

[17] Wiechmann MT, Ruzene JR, Navega MT (2013). Effects of resistive exercise in the mobility, flexibility, muscle strength, and balance of the elderly. ConScientiae Saúde.

[18] Terra R, Silva SA, Pinto VS, Dutra PM (2012). Effect of exercise on immune system: response, adaptation and cell signaling. Rev Bras Med Esporte.

[19] Vallejo R, Tilley DM, Vogel L, Benyamin R (2010). The role of glia and the immune system in the development and maintenance of neuropathic pain. Pain Pract.

[20] Miguel M, Kraychete DC, Nascimento RJ (2012). The immune system in neuropathic pain: a review. Rev Cienc Med Biol.

[21] Deschenes MR, Judelson DA, Kraemer WJ, Meskaitis VJ, Volek JS, Nindl BC (2000). Effects of resistance training on neuromuscular junction morphology. Muscle Nerve.

[22] Gosling AP (2013). Physical therapy action mechanisms and effects on pain management. Rev Dor.

[23] Schoenfeld BJ (2012). Does exercise-induced muscle damage play a role in skeletal muscle hypertrophy?. J Strength Cond Res.

[24] Bonetti LV, Schneider AP, Barbosa S, Ilha J, Faccioni-Heuser MC (2015). Balance and coordination training and endurance training after nerve injury. Muscle Nerve.

[25] Noce F, Costa VT, Simim MA, Castro HO, Samulski MD, Mello MT (2011). Analysis of overtraining symptoms during training and rehabilitation periods: a case study of Women’s Volleyball Super League team 2003/2004. Rev Bras Med Esporte.

[26] Salvini TF, Durigan JL, Peviani SM, Russo TL (2012). Effects of electrical stimulation and stretching on the adaptation of denervated skeletal muscle: implications for physical therapy. Rev Bras Fisioter.

[27] Gomes RP, Bressan E, Silva TM, Gevaerd Mda S, Tonussi CR, Domenech SC (2013). Standardization of an experimental model suitable for studies on the effect of exercise on arthritis. einstein (São Paulo).

[28] Rantanen J, Thorsson O, Wollmer P, Hurme T, Kalimo H (1999). Effects of therapeutic ultrasound on the regeneration of skeletal myofibers after experimental muscle injury. Am J Sports Med.

[29] Allen DL, Linderman JK, Roy RR, Grindeland RE, Mukku V, Edgerton VR (1997). Growth hormone/IGF-I and/or resistive exercise maintains myonuclear number in hindlimb unweighted muscles. J Appl Physiol (1985).

